# Cell type-specific regulation of CFTR trafficking—on the verge of progress

**DOI:** 10.3389/fcell.2024.1338892

**Published:** 2024-03-04

**Authors:** Carlos M. Farinha, Lúcia Santos, João F. Ferreira

**Affiliations:** Faculty of Sciences, BioISI—Biosystems and Integrative Sciences Institute, University of Lisboa, Lisboa, Portugal

**Keywords:** CFTR, protein trafficking, airway epithelium, ionocytes, ciliated cells, basal cells, secretory cells, membrane stability

## Abstract

Trafficking of the Cystic Fibrosis Transmembrane Conductance Regulator (CFTR) protein is a complex process that starts with its biosynthesis and folding in the endoplasmic reticulum. Exit from the endoplasmic reticulum (ER) is coupled with the acquisition of a compact structure that can be processed and traffic through the secretory pathway. Once reaching its final destination—the plasma membrane, CFTR stability is regulated through interaction with multiple protein partners that are involved in its post-translation modification, connecting the channel to several signaling pathways. The complexity of the process is further boosted when analyzed in the context of the airway epithelium. Recent advances have characterized in detail the different cell types that compose the surface epithelium and shifted the paradigm on which cells express CFTR and on their individual and combined contribution to the total expression (and function) of this chloride/bicarbonate channel. Here we review CFTR trafficking and its relationship with the knowledge on the different cell types of the airway epithelia. We explore the crosstalk between these two areas and discuss what is still to be clarified and how this can be used to develop more targeted therapies for CF.

## 1 Introduction

Cystic Fibrosis (CF) is caused by mutations in the gene that encodes the CF Transmembrane Conductance Regulator (CFTR) protein, which functions as a chloride and bicarbonate channel at the apical membrane of epithelial cells (Bear et al., 1992; Poulsen et al., 1994). CFTR is responsible for the regulation of the water content covering epithelia, and when defective causes the accumulation of a thick mucus in the airways (the main affected organ) leading to bacterial infections and respiratory failure.

More than 2,100 variants have been reported in the CFTR gene, over 700 of which are characterized as disease-causing. Mutations can cause CF by several mechanisms (reviewed in (Farinha, 2018)), being the major one impairment of CFTR trafficking—which is the case for p.Phe508del, the most common-disease causing mutation that leads to intracellular retention and early degradation of the mutated protein that fails to reach the plasma membrane (PM).

CFTR trafficking is a complex process and, as modulator therapies target the basic defect caused by mutations, refinement of those—to cover more mutations—and introduction of novel approaches—to target those variants that do not respond to current drugs—are challenged by understanding the mechanisms that drive CFTR expression, processing and function in the correct cell types—particularly in the airways. Recent data has revolutionized our knowledge on the airway cell types that express CFTR and on their relative contribution to its total function. In this review, we review the current knowledge on its expression in the different cell types and address the main events related to CFTR trafficking (and function) and, ending with the discussion of what is missing as we aim to address trafficking in specific cells and how this can—or needs to—be clarified to design novel—and better—therapies.

## 2 CFTR expression in the human airway epithelium

**FIGURE 1:**
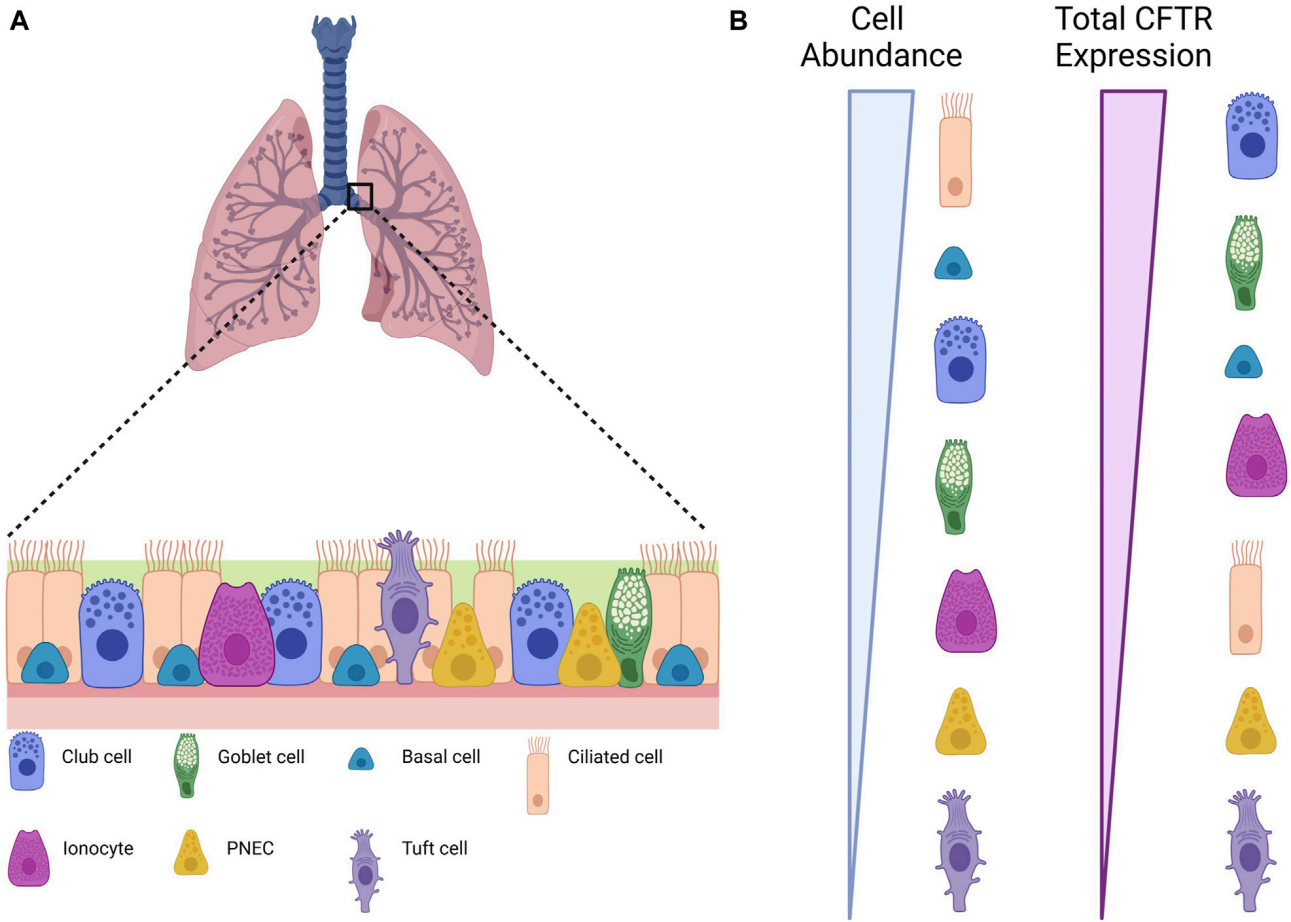
Different cell types in the airway epithelium. **(A)** Scheme depicting an airway epithelium and the different cell types that are present. **(B)** Relative CFTR expression in terms of individual cell types and of total contribution for global CFTR expression. Created with Biorender.com.

The human airway epithelium is a complex and cellularly diverse tissue that has a critical role in mucociliary clearance, the primary defense mechanism of the lungs against atmospheric contaminants, a process that is compromised in CF (Deprez et al., 2020; Goldfarbmuren et al., 2020). This pseudostratified epithelium consists of more abundant (basal, ciliated, and secretory) and rarer cell types (tuft, pulmonary neuroendocrine, and ionocytes) (Table 1; Figure 1A) (Plasschaert et al., 2018; Goldfarbmuren et al., 2020). The development of new techniques such as single-cell mRNA sequencing (scRNAseq) has helped to understand the diversity of cells present in the airway epithelium by revealing the gene expression profiles for tens of thousands of individual cells, creating a molecular cell atlas of this tissue (Montoro et al., 2018; Plasschaert et al., 2018).

**TABLE 1:** Airway cell type-enriched markers.

Cell type	Cell type-enriched marker	References
Abundant cell types		
**Basal**	*Keratin 5 (KRT5)*	Deprez et al. (2020), Goldfarbmuren et al. (2020), Travaglini et al. (2020)
	*Tumor Protein 63 (TP63)*	
Differentiating Basal	*Hes Family BHLH Transcription Factor 1 (HES1)*	
	*Keratin 7 (KRT7)*	
	*Secretoglobin Family 3A Member 2 (SCGB3A2)*	
Squamous Metaplastic	*Keratin 14 (KRT14)*	
	*Keratin 13 (KRT13)*	
**Ciliated**	*Forkhead Box J1 (FOXJ1)*	Deprez et al. (2020), Goldfarbmuren et al. (2020)
	*Tubulin Polymerization Promoting Protein Family Member 3 (TPPP3)*	
	*Sentan (SNTN)*	
Deuterosomal	*Deuterosome Assembly Protein 1 (DEUP1)*	
	*Forkhead Box N4 (FOXN4)*	
	*Cell Division Cycle 20B (CDC20B)*	
**Secretory**		Montoro et al. (2018), Plasschaert et al. (2018)
Club	*Nuclear Factor I A (NFIA)*	
	*MUC5AC*	
Goblet	*MUC5B*	
Rare cell types		
**Tuft**	*Achaete-Scute Family BHLH Transcription Factor 2 (ASCL2)*	Montoro et al. (2018), Goldfarbmuren et al. (2020)
	*POU Class 2 Homeobox 3 (POU2F3)*	
**Pulmonary Neuroendocrine (PNEC)**	*Proprotein Convertase Subtilisin/Kexin Type 1 Inhibitor (PCSK1N)*	Deprez et al. (2020)
	*Secretagogin (SCGN)*	
	*Nebulin (NEB)*	
	*Homeobox B1 (HOXB1)*	
	*Achaete-Scute Family BHLH Transcription Factor 1 (ASCL1)*	
	*Forkhead Box A2 (FOXA2)*	
**Ionocytes**	*Forkhead Box I1 (FOXI1)*	Montoro et al. (2018), Plasschaert et al. (2018), Deprez et al. (2020)
	*Cystic Fibrosis Conductance Transmembrane Regulator (CFTR)*	
	*Sodium Channel (Enac) Beta Subunit*	
	*Clc Chloride Channel Subunit Barttin (BSND)*	
	*Sodium/Potassium/Chloride Cotransporter Type 1 (NKCC1, SLC12A2)*	
	*Subunits Of The Vacuolar Proton Pump (V-Atpase)*	
	*Calcium-Activated Potassium Channel (KCNMA1)*	
	*Slc9 Family Member Of Na+/H + Exchangers (NHE7)*	
	*Cochlin (COCH)*	

### 2.1 Basal cells

Basal cells are located at the base of the epithelium and count for one-third of all cells (Travaglini and Krasnow, 2018; Deprez et al., 2020; Goldfarbmuren et al., 2020). These are progenitor cells that give rise to Club cells and to the rarer cell types (Montoro et al., 2018; Travaglini and Krasnow, 2018). Marker genes (summarized in Table 1) associated to basal cells are keratin 5 (*KRT5*) and tumor protein 63 (*TP63*), but these cells can be grouped in 3 cell populations which are distinguished by the expression of genes involved respectively in proliferation, differentiation and squamous metaplastic response to injury or stress (Deprez et al., 2020; Goldfarbmuren et al., 2020). Proliferating basal cells are enriched in the expression of genes involved in the cell cycle, indicating a proliferative state. Differentiating basal cells have a reduced expression of *KRT5* and increased expression of Hes Family BHLH Transcription Factor 1 (*HES1)*, keratin 7 (*KRT7)* and Secretoglobin Family 3A Member 2 (*SCGB3A2)*, indicating active differentiation to other epithelial fates (Travaglini et al., 2020). Examples of genes involved in the response to injury or stress are keratin 14 (*KRT14)* and keratin 13 (*KRT13)* (Goldfarbmuren et al., 2020). *CFTR* expression is low in basal cells (Montoro et al., 2018), but overall, they are the second major contributor to CFTR mRNA content in the airway epithelium (Okuda et al., 2021) (Figure 1B).

### 2.2 Ciliated cells

Ciliated cells are the most abundant airway epithelial cells in the proximal airways and have been reported as significantly decreased in CF compared with control samples (Montoro et al., 2018; Scudieri et al., 2020). Fully differentiated ciliated cells express high levels of Forkhead Box J1 (*FOXJ1)*, a transcription factor involved in ciliogenesis and ciliary maintenance (Deprez et al., 2020; Goldfarbmuren et al., 2020). Other ciliated cells markers are Tubulin Polymerization Promoting Protein Family Member 3 (*TPPP3)*, and Sentan (*SNTN)*. Deuterosomal cells, the precursors of ciliated cells, express the specific markers Deuterosome Assembly Protein 1 (*DEUP1)*, Forkhead Box N4 (*FOXN4)*, and Cell Division Cycle 20B (*CDC20B)*. These cells carry protruding structures called cilia that swirl to clear mucus and debris (Travaglini and Krasnow, 2018). Even though ciliated cells have been hypothesized to be the major source of CFTR in the proximal airway and the vastly more abundant cells, they only express 1.5% of total CFTR (Montoro et al., 2018; Plasschaert et al., 2018), positioning them among the smallest contributors to total CFTR content in the airway epithelia (Okuda et al., 2021) (Figure 1B). This apparent contradiction was clarified by a recent report showing that the strong immunofluorescence staining observed of CFTR in ciliated cells is possibly due to cross detection of the protein rootletin located at the bases of motile cilia (Sato et al., 2021).

### 2.3 Secretory cells

Club and goblet cells are both known as “secretory cells” as these 2 cell populations cluster together differing only by the levels of expression of mucin genes *MUC5AC* and *MUC5B* (Deprez et al., 2020). In fact, Club cells are known to be the precursors of Goblet and ciliated cells (Montoro et al., 2018). The Nuclear Factor I A (*NFIA)* is a transcription factor reported as enriched in Club cells (Montoro et al., 2018). This factor regulates Notch signaling, which is required for Club cell maintenance. Other markers enriched in secretory cells vary according to the maturity levels. The least-mature cells express basal cell transcripts such as *KRT5* and *TP63* while the most-mature cells express *MUC5B* (Plasschaert et al., 2018).

Secretory, as well as ciliated cells, are the 2 cell types responsible to removing inhaled particles from the upper airways (Travaglini and Krasnow, 2018; Deprez et al., 2020; Goldfarbmuren et al., 2020). Secretory cells secrete the components of the mucus, including mucins, antimicrobial and immune modulatory proteins, impeding particles from reaching deeper lung zones. Secretory cells regulate the composition of the airway surface liquid (ASL) covering the epithelium, without ASL the ciliary beating and mucociliary clearance would be compromised (Sato et al., 2023). Although CFTR levels in secretory cells are low (Montoro et al., 2018), secretory cells are the most common CFTR-expressing cells and thus they give the major contribution to total CFTR content in the airway epithelia—with a prominent role for the subtype Secretory 1 cells (Okuda et al., 2021) (Figure 1B). As secretory cells cover a large fraction of the airway surface, expression of CFTR in these cells has an important role in local control of airway surface hydration, which is in turn essential for mucus clearance.

### 2.4 Rare epithelial cells

Several studies identify low abundance clusters expressing markers enriched in Tuft cells, pulmonary neuroendocrine and ionocytes, which correspond to less than 1% of all epithelial cells (Deprez et al., 2020; Goldfarbmuren et al., 2020).

#### 2.4.1 Tuft cells

Tuft cells are suggested to be the precursors of the rare cell lineage given the similarity of Tuft-like genes expression to those of basal cells (Goldfarbmuren et al., 2020). Tuft cells-enriched markers include the Achaete-Scute Family BHLH Transcription Factor 2 (*ASCL2*) and POU Class 2 Homeobox 3 (*POU2F3*) (Montoro et al., 2018; Goldfarbmuren et al., 2020). To support the idea that Tuft cells are the precursors of the other rare cell types, Goldfarbmuren and colleagues developed a POU2F3 KO cell line that when cultured in air-liquid interface (ALI) show a significantly decrease expression in Tuft-like, ionocyte, and PNEC marker genes (Goldfarbmuren et al., 2020). Tuft cells express prostaglandin E2 (*PGE2*) and are known to have an important role in type 2 airway inflammation. The Forkhead Box I1 (*FOXI1*) transcription factor, the ionocytes marker as described below, is also detected in POU2F3+ cells however, contrary to ionocytes, Tuft cells lack detectable CFTR expression (Goldfarbmuren et al., 2020) (Figure 1B).

#### 2.4.2 Pulmonary neuroendocrine

Pulmonary neuroendocrine cells (PNECs) have a sensory function and release neuropeptides and transmitters in response to various stimuli (Sato et al., 2023). These cells express the neurotransmitter associated genes Proprotein Convertase Subtilisin/Kexin Type 1 Inhibitor (*PCSK1N)*, Secretagogin (*SCGN)*, and Nebulin (*NEB)*. The PNEC-specific regulatory units Homeobox B1 (*HOXB1*), Achaete-Scute Family BHLH Transcription Factor 1 (*ASCL1*), and Forkhead Box A2 (*FOXA2*) have also been identified as enriched in this cell type (Deprez et al., 2020). Along with neurotransmitter associated genes, the human PNECs also express voltage-gated cation channels (Deprez et al., 2020).

#### 2.4.3 Ionocytes

Pulmonary ionocytes were named after the cells with the same name found in fish gills, due to the overlap of the gene-expression profile of the 2 cell types (Travaglini and Krasnow, 2018). In fish, these cells are responsible to maintain the normal solute concentration by regulating ion (Na^+^, Cl^−^, Ca^2+^) exchange between the animal tissue and the surrounding water environment (Travaglini and Krasnow, 2018). In mammalian cells, the ionocyte function has not been clarified yet, but the expression of multiple ion-transport genes in this cell type suggests its involvement in fluid regulation at the epithelial interface (Montoro et al., 2018; Travaglini and Krasnow, 2018).

Ionocytes are characterized by the high expression of the transcription factor *FOXI1* and *CFTR* (Plasschaert et al., 2018; Deprez et al., 2020). In fact, the highest levels of CFTR expression—assessed by single cell RNAseq—occur in these low abundant cells (<0.5%), being over 500-fold higher in ionocytes than in any other cell type (Montoro et al., 2018; Goldfarbmuren et al., 2020; Sato et al., 2023) (Figure 1B). It is estimated that the ionocyte contribution to total CFTR transcripts in the airway epithelia ranges between 11% and 54%. Other studies characterized the presence and CFTR expression of ionocytes across large and small airways, and concluded, that although exhibiting high levels of CFTR expression, ionocytes contribute less to total CFTR mRNA content than secretory or basal cells (Okuda et al., 2021). Furthermore, ionocyte presence was observed as reduced in small compared to large airways, suggesting that in small airways most CFTR expression is in fact mediated by secretory cells (Okuda et al., 2021).

Other ion transporters genes enriched in ionocytes include subunits of the epithelial sodium channel (*ENaC*) beta subunit, ClC chloride channel subunit barttin (*BSND*), sodium/potassium/chloride cotransporter type 1 (*NKCC1*, *SLC12A2*), subunits of the vacuolar proton pump (V-ATPase), the calcium-activated potassium channel (*KCNMA1*) and the Slc9 family member of Na^+^/H^+^ exchangers (*NHE7*) (Plasschaert et al., 2018). *COCH* is another gene specifically expressed in ionocytes. This gene encodes a secreted protein, cochlin, that confers antibacterial activity against the two most prominent pathogens in cystic fibrosis lung disease (Montoro et al., 2018).

## 3 CFTR trafficking

### 3.1 CFTR biosynthesis and folding

CFTR biogenesis occurs through the secretory pathway—the common route through which proteins synthesized in ribosomes attached to the endoplasmic reticulum (ER) are exported to the Golgi and subsequently to their target destination in the cell, which can be the lysosomes, extracellular medium or the PM as is the case for CFTR.

As it starts emerging from ribosome, CFTR is co-translationally inserted into the ER membrane (Lu et al., 1998), due to the presence of a signal sequence of around 7–25 mainly apolar amino acid residues (von Heijne, 1985). The signal recognition particle (SRP) recognizes and binds this sequence, directing the polypeptide and the attached ribosome to the ER membrane where it binds the SRP receptor. Then, the ribosome is anchored to the translocon channel, and, after release of the SRP and the SRP receptor, the signal sequence enters this channel leading to the co-translational transfer of the polypeptide chain into the ER membrane. Simultaneously to its ER membrane insertion, the nascent CFTR undergoes glycosylation in two asparagine residues of the fourth extracellular loop. An oligosaccharide with 14 residues (containing two N-acetylglucosamine, three glucose and nine mannose residues), which is synthesized in the ER, is transferred to each asparagine residue (Enquist et al., 2009) as they are located within the N-linked glycosylation consensus sequence Asn-X-Ser/Thr (Pless and Lennarz, 1977). This glycosylation event in the ER is catalyzed by a specific oligosaccharyltransferase and it originates a core-glycosylated or immature form of the CFTR protein, also known as band B CFTR, being band A CFTR the non-glycosylated form of the protein—that can only be detected after treating CFTR with a glycosidase that trims glycans (Cheng et al., 1990). Core-glycosylated CFTR has a fast turnover rate, with a half-life of around 30 min (Lukacs et al., 1994). Additionally, the modification of immature CFTR to the mature form following the secretory pathway is not a fully efficient process (Lukacs et al., 1994). Only around 20%–40% of the newly synthesized CFTR protein acquires the native conformation (Ward and Kopito, 1994), while the remaining is rapidly degraded before exiting the ER, meaning that CFTR folding and processing is less efficient in comparison to other ABC transporter proteins (Chang et al., 1997). Nevertheless, this is dependent on cell type, as it has been reported that CFTR processing is much more efficient in human epithelial cells (Varga et al., 2004).

### 3.2 ER quality control and exit

CFTR undergoes a stringent ER quality control system (ERQC) with at least four checkpoints, which are responsible for recognizing non-native or abnormal membrane and secretory proteins and rapidly targeting them for degradation via the ubiquitin-proteasome pathway (Farinha and Amaral, 2005; Farinha et al., 2013; Canato et al., 2018). Within the ERQC machinery, molecular chaperones play a central role by facilitating correct protein folding *in vivo* through controlled binding and release of their substrate or, when this is not achieved, retaining misfolded proteins or unassembled subunits (Ohtsuka and Hata, 2000). Thus, chaperones are essential in facilitating domain assembly of CFTR and also in controlling how the glycosylation state affects CFTR trafficking.

The first checkpoint of the ERQC system occurs while the emerging CFTR polypeptide chain is being inserted into the ER membrane. During this, the exposed chain in the cytosol interacts with the cytosolic molecular chaperone Hsp70 and its co-chaperone Hdj-2 which facilitate the early steps of CFTR domain assembly and folding (Meacham, 1999; Farinha et al., 2002) and also regulate its early targeting to degradation (Kim Chiaw et al., 2019). Co-expression of Hsp70 and Hdj-1 stabilize the immature form of wt-CFTR but not of p.Phe508del-CFTR, suggesting that the amount of these molecular chaperones is not a limiting step for CFTR exit from the ER (Farinha et al., 2002)**.** For mutant p.Phe508del-CFTR, it appears to become trapped in a stronger interaction with the chaperone Hsp90 and its co-chaperone Aha1, suggesting a relevant role of this complex in its retention in the ER (Hutt et al., 2012). If CFTR is retained too long in this chaperone trap it is targeted for degradation by replacement of the productive co-chaperones with pro-degradative ones. Chaperone mediated delivery to degradation systems has been suggested to play a role in the response of CFTR variants to modulators, with more responsive mutations exhibiting decreased interactions with the proteasomal and autophagy degradation machineries (McDonald et al., 2022; Kim et al., 2023).

The second checkpoint assesses CFTR folding through its glycosylation status. The core 14-unit oligosaccharide is processed by removal of the first two glucose units by glucosidase I and this intermediate structure is recognized by chaperone lectin calnexin (and calreticulin) which allows the progress of CFTR folding (Hammond et al., 1994). The last glucose is removed by glucosidase II, decreasing affinity for calnexin (Hammond et al., 1994). If CFTR folding is unproductive, the protein becomes a target of UDP-glycoprotein glucosyltransferase, which promotes its re-glycosylation, beginning a new round of chaperone binding, de-glycosylation and folding assessment (Hammond et al., 1994). If retained for too long in this cycle, CFTR is targeted for glycoprotein-ER associated degradation (Farinha and Amaral, 2005; Farinha and Canato, 2017).

These first two checkpoints of the ERQC system occur in the early steps of CFTR biogenesis and processing, being considered ERQC folding checkpoints. Conversely, the third and fourth checkpoints are considered ERQC trafficking checkpoints. The third checkpoint involves the recognition of abnormally exposed arginine-framed tripeptides (AFTs). AFTs are transient retention/retrieval motifs, with a Arg-X-Arg sequence, that couple ER exit to the assembly and folding of multimeric membrane or secretory proteins (Zerangue et al., 1999). AFTs only act as retrieval signal when exposed in misassembled or misfolded proteins, suggesting that the motifs are buried in assembled proteins. The masking of AFTs in native proteins is the mechanism that allows these motifs to act as retrieval signals in quality control and not as constitutive retention signals (Zerangue et al., 1999). CFTR has at least four AFTs, including Arg29-Gln30-Arg31 located at the N-terminal, Arg516-Tyr517-Arg518 and Arg553-Ala554-Arg-555 located at the nucleotide binding domain 1 (NBD1) and Arg764-Ala765-Arg766 located at the regulatory domain (RD). Exposure of these motifs has been shown to lead to retention of p.Phe508del-CFTR, while simultaneous abrogation of the motifs by substitution of arginine residues 29, 516, 555, and 766 with lysine residues, allows p.Phe508del-CFTR to evade this ERQC checkpoint without acquiring the native conformation and function at the cell surface (Chang et al., 1999; Roxo-Rosa et al., 2006; Farinha et al., 2013). Additionally, it has also been shown that the introduction of these variants alters the p.Phe508del-CFTR interactome, eliciting a possible mechanism for CFTR rescue from ER retention (Canato et al., 2018).

The fourth ERQC checkpoint involves the packing of CFTR into coat complex (COP) II coated vesicles that are destined for delivery to the ER-Golgi intermediary compartment (ERGIC). This process is dependent on the interaction between COPII proteins with specific positive export signals, with the better characterized motif for CFTR being the diacidic code Asp565-Ala566-Asp567 (DAD), which is located in NBD1 (Nishimura and Balch, 1997). This motif acts as a positive cargo signal required for Sec24-mediated COPII packaging into the vesicles (Nishimura and Balch, 1997), while its disruption has been shown to decrease both association with Sec24 and exit from the ER (Wang et al., 2004) and complete abrogation has been shown to prevent CFTR processing (Farinha et al., 2013) while also partially affecting its folding (Im et al., 2023). By successfully overcoming the ERQC CFTR is finally packed into COPII coated vesicles to traffic firstly to the ERGIC and then to the Golgi apparatus.

### 3.3 Golgi processing and possible quality control

Native CFTR traffics through the different cisternae of the Golgi complex (from the early cis-Golgi to the media and then trans-Golgi) in COPI vesicles. During this process, CFTR’s ER-characteristic high-mannose glycan moieties undergo further processing by multiple Golgi glycosyltransferases and glycosidases that remove glycan units and add new ones characteristic of trans Golgi (fucose, neuraminic acid or sialic acid), forming complex structures (*in vitro* resistant to the activity of endoglycosidase H) (Amaral et al., 2016). These modifications originate a fully-glycosylated or mature form of CFTR with increased molecular weight, also known as band C CFTR (Cheng et al., 1990). After this, CFTR traffics out of the trans-Golgi for delivery at the apical PM in a process that may occur through three distinct paths: 1) directly to the membrane via transport vesicles; 2) firstly to the basolateral membrane followed by transcytosis to the apical membrane; 3) initially to apical recycling endosomes (AREs) and from there to the apical membrane. Taken together, the described processes form the conventional CFTR trafficking pathway.

In addition to this route, CFTR can traffic through possible alternative pathways from the ER to the PM, which rely on export from the ER without packaging into COPII coated vesicles or on bypassing the Golgi complex. One of the reported unconventional pathways, has been shown to occur in a cell type-specific manner, where CFTR follows a non-conventional early secretory pathway (Yoo et al., 2002). This route is insensitive to characteristic Golgi adaptors Arf1, Rab1a/Rab2 GTPases or the SNAP receptor (SNARE) component syntaxin 5. On the other hand, it depends on the late endosomal target-SNARE syntaxin 13, eliciting a possible role in CFTR maturation for recycling through a late Golgi/endosomal system (Yoo et al., 2002). These studies should however be seen with caution as they were performed in cells overexpressing CFTR. Another non-conventional secretory pathway reported is a Golgi reassembly stacking protein (GRASP)-dependent pathway, that bypasses the Golgi, and occurs only under induction of ER stress allowing the delivery of core-glycosylated CFTR, both wild type and p.Phe508del, to the PM (Gee et al., 2011). Phosphorylation of a specific site of GRASP followed by relocalization of GRASP to the ER, as well as the PDZ-based interaction of GRASP and CFTR are crucial events for this non-conventional surface trafficking pathway (Gee et al., 2011; Kim et al., 2016). More recently, another unconventional secretory pathway that also bypasses the Golgi complex has been reported for CFTR. This pathway is dependent on the activation of the IRE1α kinase-mediated signaling cascade (Park et al., 2020), which is one of the three major signaling branches of the unfolded protein response, a cellular response mechanism activated under ER stress conditions (Cox et al., 1993; Shen et al., 2001). It has been shown that IRE1α activation can promote p.Phe508del-CFTR traffic to the PM (Park et al., 2020).

### 3.4 Membrane stability—regulation by PTMs, protein-protein interactions and links to signaling pathways

**FIGURE 2:**
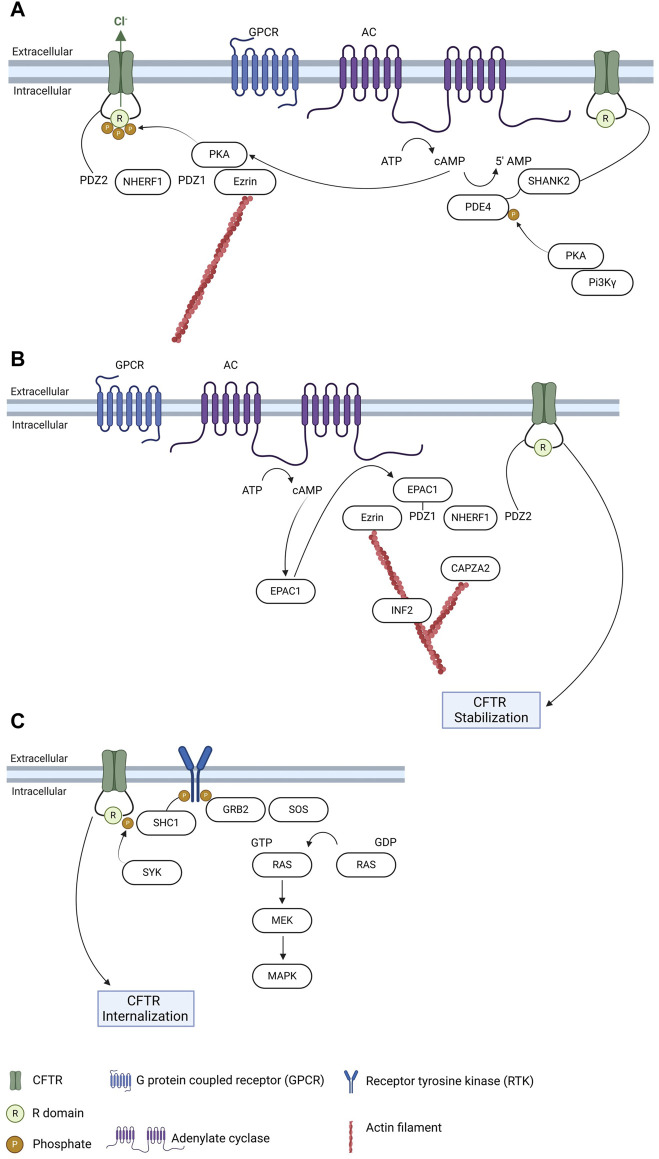
Regulation of CFTR trafficking and relation with signaling pathways. **(A)** CFTR anchoring at the plasma membrane and regulation by PDZ proteins and PKA. **(B)** Regulation of CFTR plasma membrane stability by EPAC1 and interconnections with cytoskeleton regulators. **(C)** Regulation of PM CFTR through phosphorylation by SYK and link to MAPK pathway. Created with Biorender.com.

After processing in the Golgi complex, mature CFTR is transported to the PM where it anchors. Evidence suggests that up to 50% of surface CFTR in airway epithelial cells exists in an immobile pool, tethered to filamentous actin which emphasizes the relevance of cytoskeleton structures in CFTR regulation (Haggie et al., 2006). CFTR anchoring and stability at the PM depends on the interaction with several proteins, among which PDZ-domain containing proteins (Figure 2A). These proteins, especially Na^+^/H^+^-exchanger regulatory factor isoform-1 (NHERF1), play an essential role of promoting interactions anchoring appropriate targets to the cytoskeleton through a multiprotein complex (Cushing et al., 2008). The CFTR-NHERF1 complex is locked in a static actin-tethered state through the interaction of NHERF1 with the actin-binding adaptor protein ezrin, preventing CFTR endocytosis. A role for NHERF1 has also been implicated in a diversity of processes, namely targeting exosome/endosome CFTR to the apical membrane of epithelial cells, promoting CFTR dimerization and enabling interactions that regulate CFTR conformation and activity (Short et al., 1998; Swiatecka-Urban et al., 2002; Haggie et al., 2006). While NHERF1 and NHERF2 stabilize CFTR at the PM, another PDZ-domain containing protein, CFTR-associated ligand (CAL), promotes CFTR endocytosis and degradation (Cheng et al., 2004), an interaction that has been assessed with the aim of identifying inhibitors that may prevent CFTR targeting to degradation (Zhao et al., 2018). Other proteins involved in CFTR stabilization at the PM link the channel to relevant signaling pathways in the cell, such cAMP signaling or regulation through phosphorylation by different kinases (reviewed in (Farinha et al., 2016)).

Regulation of CFTR trafficking by cAMP relies mainly on its interaction with the cAMP sensor exchange protein directly activated by cAMP 1 (EPAC1). It has been shown that EPAC1 and CFTR co-localize and interact mediated by NHERF1 in airway epithelial cells (Lobo et al., 2016). EPAC1 activation by cAMP promotes the EPAC1-NHERF1-CFTR interaction, that stabilizes the latter promoting an increase in its PM levels (Lobo et al., 2016; Ferreira et al., 2022), due to a decrease in endocytosis (Lobo et al., 2016). As EPAC1 is activated and linked to cAMP signaling, this interaction highlights a two-level regulation of the channel by cAMP—low concentrations of cAMP activate protein kinase A (PKA) to regulate CFTR function whereas high cAMP levels promote EPAC1-dependent increase of its PM levels (Lobo et al., 2016; Ferreira et al., 2022). More recently, several actin cytoskeleton dynamics regulators were identified in the macromolecular complexes elicited by EPAC1 activation at the PM (Santos et al., 2020)—among which the inverted formin 2 (INF2), a member of the formin family with the unique ability to accelerate actin filament depolymerization, in addition to the nucleation and elongation activities common to all formins (Chhabra and Higgs, 2006; Chhabra et al., 2009), and the F-actin-capping protein subunit alpha-2 (CAPZA2), which is a component of a heterodimeric CapZ protein that cap the fast growing barbed ends of actin filaments, terminating elongation (Caldwell et al., 1989; Barron-Casella et al., 1995). It has been reported that both these actin cytoskeleton regulators interact with CFTR and do not influence the EPAC1-NHERF1-CFTR interaction, suggesting that although not being key elements of the macromolecular complex they have an additional role in CFTR anchoring and stability at the PM (Santos et al., 2020) (Figure 2B).

cAMP-driven phosphorylation of CFTR triggers its association with 14-3-3 proteins, which contribute to facilitate CFTR trafficking along with enhancing its channel activity (Liang et al., 2012; Bozoky et al., 2013). cAMP effect on CFTR trafficking (and function) can also be modulated through inhibition of phosphodiesterases. By sustaining a localized pool of cAMP in CFTR vicinity, this allows an increase in CFTR levels at the PM and of its function. Selection inhibition of PDE4D—the most relevant phosphodiesterase in CFTR regulation (Blanchard et al., 2014)—with a mimetic peptide targeting the anchoring of PKA via the A-kinase anchoring protein PI3Kγ triggers CFTR function, possibly by also contributing to its PM stabilization (Ghigo et al., 2022) (Figure 2A).

Anterograde transport (traffic from the trans-Golgi to PM), endocytosis and recycling are the three mechanisms that act together to determine the amount of CFTR channels at the PM. These processes, particularly recycling and endocytosis, are finely regulated to control the levels of CFTR in the cell surface. Moreover, assessment of CFTR folding through the peripheral quality control mechanism also controls its total amount at the PM (Okiyoneda et al., 2010). An assortment of CFTR interacting proteins, namely Rab family small GTPases, PDZ domain-containing proteins and myosins, as well as specific trafficking motifs in the CFTR protein, participate in the regulation of trafficking turnover and retention of the protein at the PM.

CFTR endocytosis and internalization occurs in clathrin-coated vesicles (Bradbury et al., 1994; Lukacs et al., 1997), and is dependent on the recognition of specific motifs by endocytic adaptor proteins. These endocytic signals, usually 4-7 residue long sequences that are located in cytoplasmic domains of membrane proteins (Prince et al., 1999), are mainly localized in the C-terminus of CFTR (at least one tyrosine-based motif and one dileucine-based motif (Hu et al., 2001; Peter et al., 2002)). These signals are recognized by the μ2 subunit of the adaptor protein assembly polypeptide-2 (AP-2), and subsequently the β2 subunit, which binds to clathrin, forms a complex that initiates the CFTR internalization from the PM (Swiatecka-Urban et al., 2004; Madden and Swiatecka-Urban, 2012). After internalization, AP-2 and clathrin dissociate, recycling back to the PM, and the uncoated vesicles traffic through early endosomes (Swiatecka-Urban et al., 2004). At this stage of intracellular membrane trafficking, an intricate protein network is involved including the cytoskeleton, motor proteins and also protein kinases. The Rab and Rho subfamilies of Ras small GTPases emerge as key players in the regulation of protein vesicular transport between the different organelles of the secretory and endocytic pathways. Rab proteins are also responsible for regulating the interaction between motor proteins and cell membranes, enabling vesicle motility, and coordinating docking and fusion of vesicles with the correct target membranes. Many Rab proteins have been shown to regulate CFTR traffic through different mechanistic pathways (reviewed in (Farinha and Matos, 2018)). Rab5 regulates entry into early endosomes, from where CFTR can be rapidly recycled back to the PM in a mechanism that involves Rab4 (Gentzsch et al., 2004)**,** accounting for around 50% of internalized CFTR (Cholon et al., 2010). Additionally, CFTR can be directed to recycling endosomes, from where it can follow an alternative recycling pathway to the PM dependent on Rab11 or it can be transported to late endosomes promoted by Rab7 (Gentzsch et al., 2004). It is reported that 15%–20% of internalized CFTR remains in the recycling pool reaching a steady state (Holleran et al., 2013). A return to the trans-Golgi can also occur guided by Rab9 (Gentzsch et al., 2004; Swiatecka-Urban et al., 2005). Myosins are cytoskeletal motor proteins that also participate in CFTR trafficking through interaction with Rab proteins. Myosin VI is involved in the early endocytic stages, participating in the clustering of CFTR in clathrin-coated pits and also in the formation of clathrin-coated vesicles. This role in apical membrane endocytosis is due to movement towards the F-actin minus end, oriented away from the PM, which is uncharacteristic for most myosins (Swiatecka-Urban et al., 2004). A complex of adaptors Disabled-2 (Dab-2), AP-2 and myosin VI forms at the PM promoting actin-dependent CFTR endocytosis (Swiatecka-Urban et al., 2004; Collaco et al., 2010; Fu et al., 2015). Myosin Vb also interacts with CFTR and specifically with the isoform Rab11a, depending on the recognition of the Rab-binding sites located at its tail domain (Swiatecka-Urban et al., 2007). This interaction facilitates CFTR recycling to the apical membrane of airway epithelial cells (Swiatecka-Urban et al., 2007). The other Rab11 isoform, Rab11b, also regulates CFTR recycling to the PM but in intestinal epithelial cells, eliciting a tissue-specific role for Rab11 in CFTR trafficking (Silvis et al., 2009). Altogether, recycling of internalized CFTR to the PM is considered to be the main mechanism for sustaining a functional pool of CFTR at the cell surface.

For p.Phe508del-CFTR the amount of protein at the PM is also regulated by the peripheral quality control mechanism, which links the folding status of CFTR to its internalization. This process targets misfolded proteins that reach the PM, either naturally or through pharmacological manipulation, including rescued p.Phe508del-CFTR (Okiyoneda et al., 2010). The cytoplasmic region of conformationally unstable rescued p.Phe508del-CFTR at the PM is selectively recognized by the heat shock cognate 70 kDa protein (Hsc70), in concert with other co-chaperone proteins (Okiyoneda et al., 2010). The prolonged interaction with the Hsc70/co-chaperone complex allows the recruitment of the E3 ubiquitin ligase CHIP (C terminus of Hsc70 interacting protein), leading to ubiquitination of the conformationally defective rescued p.Phe508del-CFTR at the PM. After ubiquitination, the protein is rapidly endocytosed and signaled for delivery into the degradative lysosomal compartment (Okiyoneda et al., 2010). More recently, a mechanism that allows misfolded rescued p.Phe508del-CFTR to evade the peripheral quality control has been described (Loureiro et al., 2015). This process is dependent on a conformational change of NHERF1 which is triggered by interaction with the actin-binding adaptor protein ezrin (Loureiro et al., 2015) and can be inhibited by the protease calpain (Matos et al., 2019).

CFTR stability at the PM relies also on its interaction with membrane lipids, particularly cholesterol and sphingolipids, lipid classes whose levels are affected in CFTR-deficient cells (reviewed in (Cottrill et al., 2020)). In fact, it has been shown that CFTR clusters with membrane lipids, and that the aggregation state and dynamics of CFTR at the PM are dependent on cholesterol levels. In fact, based on cholesterol abundance at the PM, CFTR molecules can exist in two different populations—a more confined and static one in high cholesterol domains and a more abundant one that is less confined and more dynamic at lower cholesterol regions (Abu-Arish et al., 2015). These domains also bring CFTR in close association with the enzyme acid sphingomyelinase and contribute to an enhancement of transepithelial secretion with a possible role in inflammation and mucosal immunity (Abu-Arish et al., 2019). More recently, it was shown that this occurs independently of scaffolding proteins (namely PDZ proteins and actin, as described above) and is driven by lipid order (Abu-Arish et al., 2022). Interestingly, these observations on the relationship between CFTR and membrane lipids in the formation of microdomains at the PM that boost a plethora of CFTR-dependent processes are aligned with recent views and more general findings on membrane dynamics—that in fact constitute an update on the classical Singer-Nicolson fluid mosaic model for PM dynamics (Kusumi et al., 2023). Based on two of the predominant components of the PM—cholesterol and actin filaments, these CFTR-centered nanoclusters play an important role not only in “securing” its PM stability and abundance but also in the regulation of other membrane channels and transporters and in the connection to signaling pathways, as it has been shown for several other PM proteins (Saha et al., 2022; Garcia-Parajo and Mayor, 2024).

Finally, phosphorylation is a major determinant in the regulation of its trafficking (reviewed elsewhere in (Farinha et al., 2016)). In most of the cases, CFTR phosphorylation triggers interaction with specific protein adaptors—as was shown recently, e.g., for the mechanism of CFTR PM regulation by spleen tyrosine kinase (SYK). Known for long to regulate CFTR PM levels (Luz et al., 2011; Mendes et al., 2011), SYK phosphorylates CFTR prompting recognition by the adaptor protein SHC1, a key regulator of MAPK pathway activity (Loureiro et al., 2020; Barros et al., 2023) (Figure 2C).

The intricate mechanisms that govern CFTR trafficking have been elucidated using a plethora of cellular models, each of which gave relevant contributions to the overall picture but that may be difficult to integrate. The challenge has increased significantly with recent advances in identification of all the cell types in the airway epithelium and their relative contribution to total CFTR expression.

## 4 Cell type-specific regulation of CFTR

As mentioned above, the highest levels of CFTR expression in the airway epithelium are detected in pulmonary ionocytes. Consequently, understanding the regulation of CFTR in this cell type would be critical for the development of new therapeutical approaches for CF targeting either expression/trafficking or function of CFTR.

However, the role of CFTR in pulmonary ionocytes is still poorly understood. It has been demonstrated that FOXI1 overexpression is sufficient to specify ionocytes-enriched cultures and, in agreement, FOXI1 KO leads to cultures lacking ionocytes (Montoro et al., 2018; Plasschaert et al., 2018). As these cells express high levels of CFTR, a decrease in this gene would be expected in FOXI1 KO cultures. However, the bulk CFTR expression is unaltered in FOXI1 knock-down (KD) cultures, suggesting that other epithelial cell types contribute to the overall CFTR expression (Goldfarbmuren et al., 2020), namely secretory and basal cells identified as the major contributors to total CFTR content (Okuda et al., 2021). However, FOXI1 KD ALI cultures showed increased transmembrane potential along with decreased conductance, which is consistent with a reduction in ion transport in ionocyte-depleted epithelia, suggesting that ionocytes contribute to the maintenance of proper ion transport (Goldfarbmuren et al., 2020). This observation confirms that while having a great contribution to CFTR expression and being critical for ion transport homeostasis, CFTR activity in the human airway epithelium is not exclusive from ionocytes. Altogether, these data raise the possibility of a cell type-specific regulation of CFTR (Sato et al., 2023).

**FIGURE 3:**
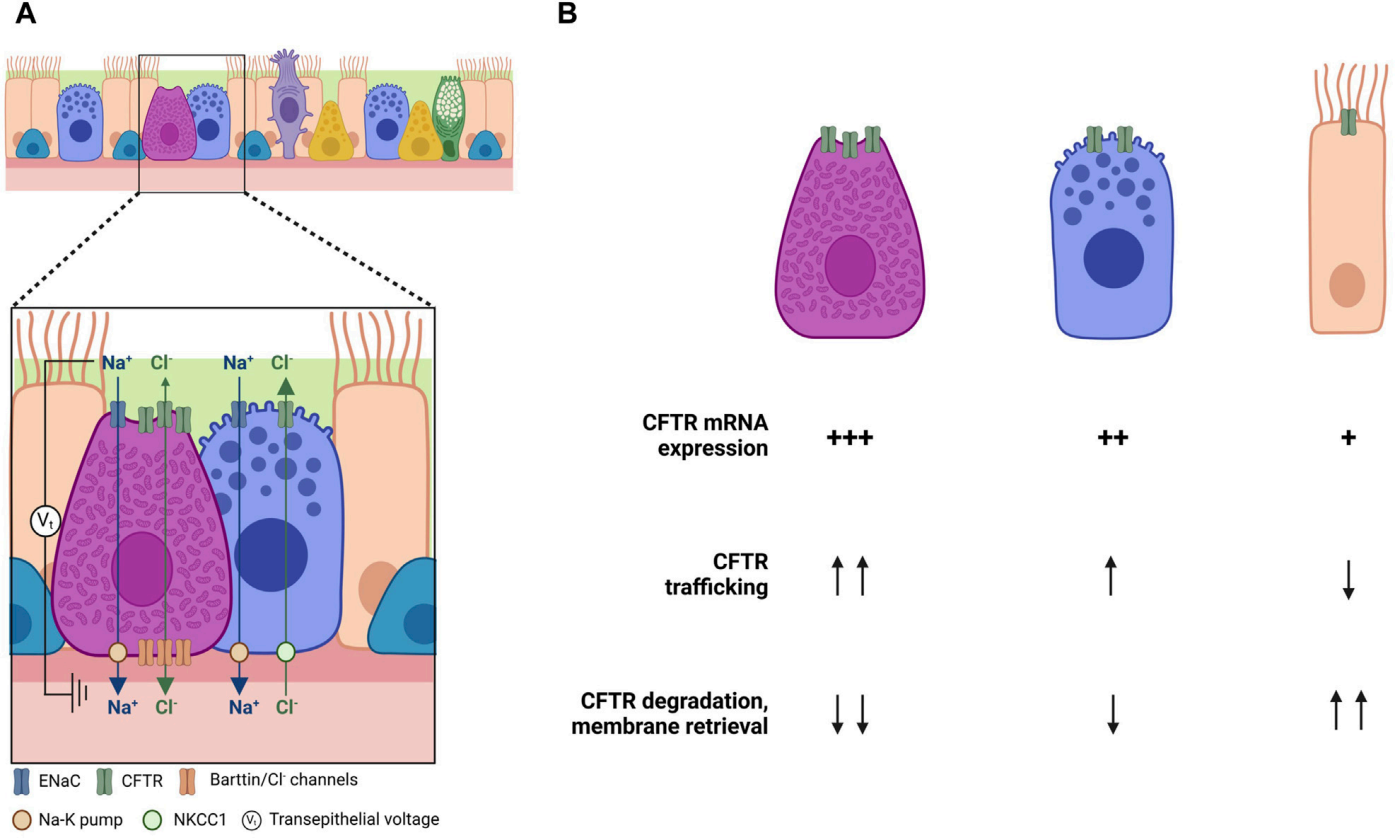
Cell-type specific regulation of CFTR function and trafficking. **(A)** Regulation of Cl^−^ transport by differential cellular roles in the airways (adapted from Tümmler, 2023). **(B)** Possible explanations for the differences on CFTR expression and trafficking among cell types. Created with Biorender.com.

Another study shows contradictory results in ionocytes. High levels of CFTR expression in ionocytes would suggest an increase in the volume of ASL due to ionocyte liquid secretion. Nonetheless, Lei and coworkers observed an increase in liquid absorption, and consequently a decrease in ASL volume in ionocyte-rich cultures, while a reduction in ionocyte abundance led to an increased liquid secretion (Lei et al., 2023). A possible explanation for that is the presence of basolateral barttin/Cl^−^ channels in ionocytes. Barttin, as previously mentioned, is a required subunit of human ClC-K Cl^−^ channels, whose expression is enriched in ionocytes. Disruption of the *BSND* gene, which encodes barttin, increased the ASL volume and decreased liquid absorption, indicating that ionocytes require barttin/Cl^−^ channels for apical-to-basolateral Cl^−^ flux and ASL absorption. These data suggests that ionocytes provide a pathway for passive Cl^−^ absorption through apical CFTR channels and basolateral barttin/Cl^−^ channels (Figure 3A). The major driving force for Cl^−^ absorption is the negative luminal voltage generated by Na^+^ absorption. In fact, apical epithelial Na^+^ channels (ENaC) inhibition by amiloride abolished the effect of ionocytes on liquid absorption. Conversely, secretory cells might provide a pathway for active Cl^−^ secretion through apical CFTR channels. This idea is supported by the fact that secretory cells lack barttin/ClC-K channels, and when barttin/ClC-K channels are overexpressed in these cells, liquid absorption increases. Once again, these data suggest that CFTR is differentially regulated, with secretion and absorption segregating between different cell types—with ionocytes involved mainly in absorption and secretory and basal cells mainly in secretion (Tümmler, 2023).

Studies performed in ferrets have further contributed to the elucidation of ionocytes role. Using conditional genetics allowed the identification of three subtypes of pulmonary ionocytes and the confirmation of their CFTR-dependent function. In fact, this work highlights the role of ionocytes in supporting a movement of Cl^−^ and HCO_3_^−^ that was found to be cell-autonomous, thus providing unique layers in the regulation of ASL volume, PH and viscosity, thus contributing to effective mucociliary clearance (Yuan et al., 2023).

CFTR regulation on the different epithelia cell types has been the research focus of many research groups, and ionocytes are now in the spotlight given their high CFTR expression. CFTR channel activation is regulated by cAMP. Briefly, as a response to an external stimulus, adenylyl cyclase is activated, increasing the levels of cAMP inside the cell (Sheppard and Welsh, 1999; Moran, 2010). cAMP activates protein kinase A (PKA), which then causes the phosphorylation of serine residues within the RD, activating CFTR (Sheppard and Welsh, 1999; Moran, 2010). Then, ATP binds to both NBDs promoting their dimerization and ATP hydrolysis (Sheppard and Welsh, 1999; Moran, 2010). This process leads to a conformational change in the membrane-spanning domains (MSDs) of CFTR, which allow Cl^−^ anions to flow through (Sheppard and Welsh, 1999; Moran, 2010). Sato and colleagues showed that in ionocyte-rich cultures CFTR regulation by cAMP is altered (Sato et al., 2023). Stimulation of ionocyte-rich cultures with high doses of the adenylyl cyclase activator forskolin did not increase total intracellular cAMP and, consequently, did not increase CFTR response. Levels of adenylyl cyclase 5 (AC5) transcripts were found increased in ionocyte-rich cultures. AC5 is inhibited by PKA phosphorylation, which is then susceptible to a feedback inhibition at high cAMP concentration, limiting CFTR response (Sato et al., 2023).

The same authors found a 10-fold increase in the Phosphodiesterase 1C (PDE1C) transcript levels in ionocyte-rich cultures. PDE1C is a calcium/calmodulin-dependent enzyme with high affinity for cAMP. Inhibition of PDE1C led to an increase in global cAMP levels and to an increase in short-circuit current (I_sc_), suggesting that PDE1C modulates cAMP signaling and CFTR activity in ionocytes. PDE1C inhibition with IBMX (that inhibits all PDEs except PDE8) and PF-04957325 (PDE8 inhibitor) further increased I_sc_ compared to inhibition using IBMX alone, suggesting that CFTR activity is modulated by different PDEs in different cell-types. In fact, other PDE family members, such as PDE3, 4 (see above) and 5, have already been implicated in CFTR regulation and the use of PDE inhibitors has already been proposed as a potential therapy for CF (Turner et al., 2021).

Focus on ionocyte development in CF and the relationship between ionocytes and CFTR-dependent chloride secretion identified a relatively high expression of ionocyte markers in the nasal epithelium with a possible proximal-distal gradient along the airways with a progressively decreasing number of ionocytes (Scudieri et al., 2020), a finding also reported by others (Okuda et al., 2021). Nonetheless, no difference was observed in the percentage of ionocytes (FOXI1+ cells) in the nasal epithelium of people with CF (PwCF) compared to controls, suggesting that the lack of functional CFTR does not affect the abundance of these cells. To understand if there was a relationship between the CFTR genotype and CFTR protein expression in ionocytes, CFTR signal in the apical membrane of ionocytes was compared in cells from non-CF individuals with cells from two groups of CF individuals. One group included individuals with CF and severe mutations (p.Phe508del, p.Glu585Ter, p.Asn1303Lys, deletions, and frameshift mutations), while the other group included those individuals with milder mutations (p.Asp1152His, p.ArgR117His, or the 5T/12 TG polymorphism). Interestingly, CFTR signal was detected at the apical membrane of ionocytes from individuals with milder mutations. Despite being lower than the signal in non-CF cells, that signal was significantly higher than that in cells from individuals with severe mutations. Assessment of the efficacy of CFTR modulators to rescue mutant CFTR protein in ionocytes showed that the two correctors, VX-809 and VX-445, markedly increased CFTR function in bronchial epithelial cells from p.Phe508del homozygous patients and that the corrector treatment improved CFTR trafficking in ionocytes.

And what about cell-type specific regulation of CFTR processing and trafficking? Recent years have seen enormous progress in the elucidation of the cell types that express CFTR and of their relative contributions to total content (and perhaps) function. Some light is appearing over puzzling observations, suggesting a differential role for CFTR in promoting either Cl^−^ secretion or absorption (Tümmler, 2023). However, very little (or nothing) is known about how CFTR traffics to the cell surface, and how the differences in individual content are not only related to differences in CFTR mRNA levels but also in mRNA transcription rates and stability, in CFTR translation, in its membrane integration and folding, its trafficking through the Golgi or its PM stability (Figure 3B).

Another relevant question is the targeting of individual cell types. Although approval of modulators has revolutionized the CF field, many individuals with CF bear genotypes that cannot be treated with the approved drugs. Furthermore, inequalities in access decrease the proportion of those that are under modulator therapy to only 12% of estimated total of individuals with CF worldwide (Guo et al., 2022). Thus, it is crucial to bring novel (and more accessible and affordable) therapies to the CF community and to target relevant cells.

When considering genetic-based therapies (cDNA, mRNA or gene editing), three main aspects need to be considered: 1) the cargo to be delivered; 2) the delivery methodology (e.g. viral vs. non-viral) and 3) the cells to be targeted. The “obvious” target would be basal cells, due to their ability to differentiate into the other cell types. However, their location at the basis of the pseudostratified epithelium makes them difficult to target. Additionally, basal cell activation requires injury to the surface epithelium to trigger their migration and proliferation, and basal cell replication is much lower in CF than in healthy lung (Carraro et al., 2021). Another hypothesis is thus to target specific cells. Ionocytes could be a candidate—due to their high expression of CFTR, but recent findings of their differential role (Tümmler, 2023) bring additional complexity to the question. Regarding other cell types, it has been shown that secretory cells are competent to correct CFTR function in CF cells (Okuda et al., 2021), which is a very relevant observation. Despite these advances, there are however many questions still to be solved in this quest.

Finally, as the puzzle comes together, it will be relevant to clarify if the balance of the different cell types (“individual” CFTR expression *versus* contribution to total CFTR content) can be changed and whether this will lead or not to a better correction.

## References

[B1] Abu-Arish A. Pandzic E. Goepp J. Matthes E. Hanrahan J. W. Wiseman P. W. (2015). Cholesterol modulates CFTR confinement in the plasma membrane of primary epithelial cells. Biophys. J. 109, 85–94. 10.1016/j.bpj.2015.04.042 26153705 PMC4572494

[B2] Abu-Arish A. Pandžić E. Kim D. Tseng H. W. Wiseman P. W. Hanrahan J. W. (2019). Agonists that stimulate secretion promote the recruitment of CFTR into membrane lipid microdomains. J. Gen. Physiol. 151, 834–849. 10.1085/jgp.201812143 31048413 PMC6572005

[B3] Abu-Arish A. Pandžić E. Luo Y. Sato Y. Turner M. J. Wiseman P. W. (2022). Lipid-driven CFTR clustering is impaired in cystic fibrosis and restored by corrector drugs. J. Cell Sci. 135, jcs259002. 10.1242/jcs.259002 35060604 PMC8976878

[B4] Amaral M. D. Farinha C. M. Matos P. Botelho H. M. (2016). Investigating alternative transport of integral plasma membrane proteins from the ER to the Golgi: lessons from the cystic fibrosis transmembrane conductance regulator (CFTR). Methods Mol. Biol. 1459, 105–126. 10.1007/978-1-4939-3804-9_7 27665554

[B5] Barron-Casella E. A. Torres M. A. Scherer S. W. Heng H. H. Q. Tsui L.-C. Casella J. F. (1995). Sequence analysis and chromosomal localization of human Cap Z. Conserved residues within the actin-binding domain may link Cap Z to gelsolin/severin and profilin protein families. J. Biol. Chem. 270, 21472–21479. 10.1074/jbc.270.37.21472 7665558

[B6] Barros P. Matos A. M. Matos P. Jordan P. (2023). YES1 kinase mediates the membrane removal of rescued F508del-CFTR in airway cells by promoting MAPK pathway activation via SHC1. Biomolecules 13, 949. 10.3390/biom13060949 37371529 PMC10296715

[B7] Bear C. E. Li C. Kartner N. Bridges R. J. Jensen T. J. Ramjeesingh M. (1992). Purification and functional reconstitution of the cystic fibrosis transmembrane conductance regulator (CFTR). Cell 68, 809–818. 10.1016/0092-8674(92)90155-6 1371239

[B8] Blanchard E. Zlock L. Lao A. Mika D. Namkung W. Xie M. (2014). Anchored PDE4 regulates chloride conductance in wild‐type and ΔF508‐CFTR human airway epithelia. FASEB J. 28, 791–801. 10.1096/fj.13-240861 24200884 PMC3898646

[B9] Bozoky Z. Krzeminski M. Muhandiram R. Birtley J. R. Al-Zahrani A. Thomas P. J. (2013). Regulatory R region of the CFTR chloride channel is a dynamic integrator of phospho-dependent intra- and intermolecular interactions. Proc. Natl. Acad. Sci. 110, E4427–E4436. 10.1073/pnas.1315104110 24191035 PMC3839774

[B10] Bradbury N. A. Cohn J. A. Venglarik C. J. Bridges R. J. (1994). Biochemical and biophysical identification of cystic fibrosis transmembrane conductance regulator chloride channels as components of endocytic clathrin-coated vesicles. J. Biol. Chem. 269, 8296–8302. 10.1016/S0021-9258(17)37192-2 7510684

[B11] Caldwell J. E. Heiss S. G. Mermall V. Cooper J. A. (1989). Effects of CapZ, an actin-capping protein of muscle, on the polymerization of actin. Biochemistry 28, 8506–8514. 10.1021/bi00447a036 2557904

[B12] Canato S. Santos J. D. Carvalho A. S. Aloria K. Amaral M. D. Matthiesen R. (2018). Proteomic interaction profiling reveals KIFC1 as a factor involved in early targeting of F508del-CFTR to degradation. Cell. Mol. Life Sci. 75, 4495–4509. 10.1007/s00018-018-2896-7 30066085 PMC11105581

[B13] Carraro G. Langerman J. Sabri S. Lorenzana Z. Purkayastha A. Zhang G. (2021). Transcriptional analysis of cystic fibrosis airways at single-cell resolution reveals altered epithelial cell states and composition. Nat. Med. 27, 806–814. 10.1038/s41591-021-01332-7 33958799 PMC9009537

[B14] Chang X. Cui L. Hou Y. Jensen T. J. Aleksandrov A. A. Mengos A. (1999). Removal of multiple arginine-framed trafficking signals overcomes misprocessing of ΔF508 CFTR present in most patients with cystic fibrosis. Mol. Cell 4, 137–142. 10.1016/S1097-2765(00)80196-3 10445036

[B15] Chang X.-B. Hou Y.-X. Riordan J. R. (1997). ATPase activity of purified multidrug resistance-associated protein. J. Biol. Chem. 272, 30962–30968. 10.1074/jbc.272.49.30962 9388243

[B16] Cheng J. Wang H. Guggino W. B. (2004). Modulation of mature cystic fibrosis transmembrane regulator protein by the PDZ domain protein CAL. J. Biol. Chem. 279, 1892–1898. 10.1074/jbc.M308640200 14570915

[B17] Cheng S. H. Gregory R. J. Marshall J. Paul S. Souza D. W. White G. A. (1990). Defective intracellular transport and processing of CFTR is the molecular basis of most cystic fibrosis. Cell 63, 827–834. 10.1016/0092-8674(90)90148-8 1699669

[B18] Chhabra E. S. Higgs H. N. (2006). INF2 is a WASP homology 2 motif-containing formin that severs actin filaments and accelerates both polymerization and depolymerization. J. Biol. Chem. 281, 26754–26767. 10.1074/jbc.M604666200 16818491

[B19] Chhabra E. S. Ramabhadran V. Gerber S. A. Higgs H. N. (2009). INF2 is an endoplasmic reticulum-associated formin protein. J. Cell Sci. 122, 1430–1440. 10.1242/jcs.040691 19366733 PMC2721004

[B20] Cholon D. M. O’Neal W. K. Randell S. H. Riordan J. R. Gentzsch M. (2010). Modulation of endocytic trafficking and apical stability of CFTR in primary human airway epithelial cultures. Am. J. Physiol. Cell. Mol. Physiol. 298, L304–L314. 10.1152/ajplung.00016.2009 PMC2838667 20008117

[B21] Collaco A. Jakab R. Hegan P. Mooseker M. Ameen N. (2010). Alpha-AP-2 directs myosin VI-dependent endocytosis of cystic fibrosis transmembrane conductance regulator chloride channels in the intestine. J. Biol. Chem. 285, 17177–17187. 10.1074/jbc.M110.127613 20351096 PMC2878018

[B22] Cottrill K. A. Farinha C. M. McCarty N. A. (2020). The bidirectional relationship between CFTR and lipids. Commun. Biol. 3, 179. 10.1038/s42003-020-0909-1 32313074 PMC7170930

[B23] Cox J. S. Shamu C. E. Walter P. (1993). Transcriptional induction of genes encoding endoplasmic reticulum resident proteins requires a transmembrane protein kinase. Cell 73, 1197–1206. 10.1016/0092-8674(93)90648-A 8513503

[B24] Cushing P. R. Fellows A. Villone D. Boisguérin P. Madden D. R. (2008). The relative binding affinities of PDZ partners for CFTR: a biochemical basis for efficient endocytic recycling. Biochemistry 47, 10084–10098. 10.1021/bi8003928 18754678 PMC2582146

[B25] Deprez M. Zaragosi L.-E. Truchi M. Becavin C. Ruiz García S. Arguel M.-J. (2020). A single-cell atlas of the human healthy airways. Am. J. Respir. Crit. Care Med. 202, 1636–1645. 10.1164/rccm.201911-2199OC 32726565

[B26] Enquist K. Fransson M. Boekel C. Bengtsson I. Geiger K. Lang L. (2009). Membrane-integration characteristics of two ABC transporters, CFTR and P-glycoprotein. J. Mol. Biol. 387, 1153–1164. 10.1016/j.jmb.2009.02.035 19236881

[B27] Farinha C. M. (2018). CFTR and cystic fibrosis. 1st ed. Cham: Springer International Publishing. 10.1007/978-3-319-65494-2

[B28] Farinha C. M. Amaral M. D. (2005). Most F508del-CFTR is targeted to degradation at an early folding checkpoint and independently of calnexin. Mol. Cell. Biol. 25, 5242–5252. 10.1128/MCB.25.12.5242-5252.2005 15923638 PMC1140594

[B29] Farinha C. M. Canato S. (2017). From the endoplasmic reticulum to the plasma membrane: mechanisms of CFTR folding and trafficking. Cell. Mol. Life Sci. 74, 39–55. 10.1007/s00018-016-2387-7 27699454 PMC11107782

[B30] Farinha C. M. King-Underwood J. Sousa M. Correia A. R. Henriques B. J. Roxo-Rosa M. (2013). Revertants, low temperature, and correctors reveal the mechanism of F508del-CFTR rescue by VX-809 and suggest multiple agents for full correction. Chem. Biol. 20, 943–955. 10.1016/j.chembiol.2013.06.004 23890012

[B31] Farinha C. M. Matos P. (2018). Rab GTPases regulate the trafficking of channels and transporters – a focus on cystic fibrosis. Small GTPases 9, 136–144. 10.1080/21541248.2017.1317700 28463591 PMC5902203

[B32] Farinha C. M. Nogueira P. Mendes F. Penque D. Amaral M. D. (2002). The human DnaJ homologue (Hdj)-1/heat-shock protein (Hsp) 40 co-chaperone is required for the *in vivo* stabilization of the cystic fibrosis transmembrane conductance regulator by Hsp70. Biochem. J. 366, 797–806. 10.1042/bj20011717 12069690 PMC1222832

[B33] Farinha C. M. Swiatecka-Urban A. Brautigan D. L. Jordan P. (2016). Regulatory crosstalk by protein kinases on CFTR trafficking and activity. Front. Chem. 4, 1. 10.3389/fchem.2016.00001 26835446 PMC4718993

[B34] Ferreira J. F. Silva I. A. L. Botelho H. M. Amaral M. D. Farinha C. M. (2022). Absence of EPAC1 signaling to stabilize CFTR in intestinal organoids. Cells 11, 2295. 10.3390/cells11152295 35892592 PMC9332071

[B35] Fu L. Rab A. Tang L. P. Bebok Z. Rowe S. M. Bartoszewski R. (2015). ΔF508 CFTR surface stability is regulated by DAB2 and CHIP-mediated ubiquitination in post-endocytic compartments. PLoS One 10, e0123131. 10.1371/journal.pone.0123131 25879443 PMC4399842

[B36] Garcia-Parajo M. F. Mayor S. (2024). The ubiquitous nanocluster: a molecular scale organizing principle that governs cellular information flow. Curr. Opin. Cell Biol. 86, 102285. 10.1016/j.ceb.2023.102285 38056142 PMC7617173

[B37] Gee H. Y. Noh S. H. Tang B. L. Kim K. H. Lee M. G. (2011). Rescue of Δf508-CFTR trafficking via a GRASP-dependent unconventional secretion pathway. Cell 146, 746–760. 10.1016/j.cell.2011.07.021 21884936

[B38] Gentzsch M. Chang X.-B. Cui L. Wu Y. Ozols V. V. Choudhury A. (2004). Endocytic trafficking routes of wild type and DeltaF508 cystic fibrosis transmembrane conductance regulator. Mol. Biol. Cell 15, 2684–2696. 10.1091/mbc.e04-03-0176 15075371 PMC420093

[B39] Ghigo A. Murabito A. Sala V. Pisano A. R. Bertolini S. Gianotti A. (2022). A PI3Kγ mimetic peptide triggers CFTR gating, bronchodilation, and reduced inflammation in obstructive airway diseases. Sci. Transl. Med. 14, eabl6328. 10.1126/scitranslmed.abl6328 35353541 PMC9869178

[B40] Goldfarbmuren K. C. Jackson N. D. Sajuthi S. P. Dyjack N. Li K. S. Rios C. L. (2020). Dissecting the cellular specificity of smoking effects and reconstructing lineages in the human airway epithelium. Nat. Commun. 11, 2485. 10.1038/s41467-020-16239-z 32427931 PMC7237663

[B41] Guo J. Garratt A. Hill A. (2022). Worldwide rates of diagnosis and effective treatment for cystic fibrosis. J. Cyst. Fibros. 21, 456–462. 10.1016/j.jcf.2022.01.009 35125294

[B42] Haggie P. M. Kim J. K. Lukacs G. L. Verkman A. S. (2006). Tracking of quantum dot-labeled CFTR shows near immobilization by C-terminal PDZ interactions. Mol. Biol. Cell 17, 4937–4945. 10.1091/mbc.e06-08-0670 16987954 PMC1679663

[B43] Hammond C. Braakman I. Helenius A. (1994). Role of N-linked oligosaccharide recognition, glucose trimming, and calnexin in glycoprotein folding and quality control. Proc. Natl. Acad. Sci. 91, 913–917. 10.1073/pnas.91.3.913 8302866 PMC521423

[B44] Holleran J. P. Zeng J. Frizzell R. A. Watkins S. C. (2013). Regulated recycling of mutant CFTR is partially restored by pharmacological treatment. J. Cell Sci. 126, 2692–2703. 10.1242/jcs.120196 23572510 PMC3687701

[B45] Hu W. Howard M. Lukacs G. L. (2001). Multiple endocytic signals in the C-terminal tail of the cystic fibrosis transmembrane conductance regulator. Biochem. J. 354, 561–572. 10.1042/0264-6021:3540561 11237860 PMC1221687

[B46] Hutt D. M. Roth D. M. Chalfant M. A. Youker R. T. Matteson J. Brodsky J. L. (2012). FK506 binding protein 8 peptidylprolyl isomerase activity manages a late stage of cystic fibrosis transmembrane conductance regulator (CFTR) folding and stability. J. Biol. Chem. 287, 21914–21925. 10.1074/jbc.M112.339788 22474283 PMC3381152

[B47] Im J. Hillenaar T. Yeoh H. Y. Sahasrabudhe P. Mijnders M. van Willigen M. (2023). ABC-transporter CFTR folds with high fidelity through a modular, stepwise pathway. Cell. Mol. Life Sci. 80, 33. 10.1007/s00018-022-04671-x 36609925 PMC9825563

[B48] Kim J. Noh S. H. Piao H. Kim D. H. Kim K. Cha J. S. (2016). Monomerization and ER relocalization of GRASP is a requisite for unconventional secretion of CFTR. Traffic 17, 733–753. 10.1111/tra.12403 27062250

[B49] Kim M. McDonald E. F. Sabusap C. M. P. Timalsina B. Joshi D. Hong J. S. (2023). Elexacaftor/VX-445–mediated CFTR interactome remodeling reveals differential correction driven by mutation-specific translational dynamics. J. Biol. Chem. 299, 105242. 10.1016/j.jbc.2023.105242 37690692 PMC10579539

[B50] Kim Chiaw P. Hantouche C. Wong M. J. H. Matthes E. Robert R. Hanrahan J. W. (2019). Hsp70 and DNAJA2 limit CFTR levels through degradation. PLoS One 14, e0220984. 10.1371/journal.pone.0220984 31408507 PMC6692068

[B51] Kusumi A. Tsunoyama T. A. Tang B. Hirosawa K. M. Morone N. Fujiwara T. K. (2023). Cholesterol- and actin-centered view of the plasma membrane: updating the Singer–Nicolson fluid mosaic model to commemorate its 50th anniversary †. Mol. Biol. Cell 34, pl1. 10.1091/mbc.E20-12-0809 37039596 PMC10162409

[B52] Lei L. Traore S. Romano Ibarra G. S. Karp P. H. Rehman T. Meyerholz D. K. (2023). CFTR-rich ionocytes mediate chloride absorption across airway epithelia. J. Clin. Invest. 133, e171268. 10.1172/JCI171268 37581935 PMC10575720

[B53] Liang X. Da Paula A. C. Bozóky Z. Zhang H. Bertrand C. A. Peters K. W. (2012). Phosphorylation-dependent 14-3-3 protein interactions regulate CFTR biogenesis. Mol. Biol. Cell 23, 996–1009. 10.1091/mbc.e11-08-0662 22278744 PMC3302758

[B54] Lobo M. J. Amaral M. D. Zaccolo M. Farinha C. M. (2016). EPAC1 activation by cAMP stabilizes CFTR at the membrane by promoting its interaction with NHERF1. J. Cell Sci. 129, 2599–2612. 10.1242/jcs.185629 27206858

[B55] Loureiro C. A. Matos A. M. Dias-Alves Â. Pereira J. F. Uliyakina I. Barros P. (2015). A molecular switch in the scaffold NHERF1 enables misfolded CFTR to evade the peripheral quality control checkpoint. Sci. Signal. 8, ra48. 10.1126/scisignal.aaa1580 25990958

[B56] Loureiro C. A. Pinto F. R. Barros P. Matos P. Jordan P. (2020). A SYK/SHC1 pathway regulates the amount of CFTR in the plasma membrane. Cell. Mol. Life Sci. 77, 4997–5015. 10.1007/s00018-020-03448-4 31974654 PMC11105000

[B57] Lu Y. Xiong X. Helm A. Kimani K. Bragin A. Skach W. R. (1998). Co- and posttranslational translocation mechanisms direct cystic fibrosis transmembrane conductance regulator N terminus transmembrane assembly. J. Biol. Chem. 273, 568–576. 10.1074/jbc.273.1.568 9417117

[B58] Lukacs G. L. Mohamed A. Kartner N. Chang X. B. Riordan J. R. Grinstein S. (1994). Conformational maturation of CFTR but not its mutant counterpart (delta F508) occurs in the endoplasmic reticulum and requires ATP. EMBO J. 13, 6076–6086. 10.1002/j.1460-2075.1994.tb06954.x 7529176 PMC395586

[B59] Lukacs G. L. Segal G. Kartner N. Grinstein S. Zhang F. (1997). Constitutive internalization of cystic fibrosis transmembrane conductance regulator occurs via clathrin-dependent endocytosis and is regulated by protein phosphorylation. Biochem. J. 328 (2), 353–361. 10.1042/bj3280353 9371688 PMC1218928

[B60] Luz S. Kongsuphol P. Mendes A. I. Romeiras F. Sousa M. Schreiber R. (2011). Contribution of casein kinase 2 and spleen tyrosine kinase to CFTR trafficking and protein kinase A-induced activity. Mol. Cell. Biol. 31, 4392–4404. 10.1128/MCB.05517-11 21930781 PMC3209257

[B61] Madden D. R. Swiatecka-Urban A. (2012). Tissue-specific control of CFTR endocytosis by Dab2: cargo recruitment as a therapeutic target. Commun. Integr. Biol. 5, 473–476. 10.4161/cib.21375 23181163 PMC3502210

[B62] Matos A. M. Pinto F. R. Barros P. Amaral M. D. Pepperkok R. Matos P. (2019). Inhibition of calpain 1 restores plasma membrane stability to pharmacologically rescued Phe508del-CFTR variant. J. Biol. Chem. 294, 13396–13410. 10.1074/jbc.RA119.008738 31324722 PMC6737230

[B63] McDonald E. F. Sabusap C. M. P. Kim M. Plate L. (2022). Distinct proteostasis states drive pharmacologic chaperone susceptibility for cystic fibrosis transmembrane conductance regulator misfolding mutants. Mol. Biol. Cell 33, ar62. 10.1091/mbc.E21-11-0578 35389766 PMC9561855

[B64] Meacham G. C. Lu Z. King S. Sorscher E. Tousson A. Cyr D. M. (1999). The Hdj-2/Hsc70 chaperone pair facilitates early steps in CFTR biogenesis. EMBO J. 18, 1492–1505. 10.1093/emboj/18.6.1492 10075921 PMC1171238

[B65] Mendes A. I. Matos P. Moniz S. Luz S. Amaral M. D. Farinha C. M. (2011). Antagonistic regulation of cystic fibrosis transmembrane conductance regulator cell surface expression by protein kinases WNK4 and spleen tyrosine kinase. Mol. Cell. Biol. 31, 4076–4086. 10.1128/MCB.05152-11 21807898 PMC3187369

[B66] Montoro D. T. Haber A. L. Biton M. Vinarsky V. Lin B. Birket S. E. (2018). A revised airway epithelial hierarchy includes CFTR-expressing ionocytes. Nature 560, 319–324. 10.1038/s41586-018-0393-7 30069044 PMC6295155

[B67] Moran O. (2010). Model of the cAMP activation of chloride transport by CFTR channel and the mechanism of potentiators. J. Theor. Biol. 262, 73–79. 10.1016/j.jtbi.2009.08.032 19766125

[B68] Nishimura N. Balch W. E. (1997). A di-acidic signal required for selective export from the endoplasmic reticulum. Sci. (80-) 277, 556–558. 10.1126/science.277.5325.556 9228004

[B69] Ohtsuka K. Hata M. (2000). Molecular chaperone function of mammalian Hsp70 and Hsp40-a review. Int. J. Hyperth. 16, 231–245. 10.1080/026567300285259 10830586

[B70] Okiyoneda T. Barrière H. Bagdány M. Rabeh W. M. Du K. Höhfeld J. (2010). Peripheral protein quality control removes unfolded CFTR from the plasma membrane. Sci. (80-) 329, 805–810. 10.1126/science.1191542 PMC5026491 20595578

[B71] Okuda K. Dang H. Kobayashi Y. Carraro G. Nakano S. Chen G. (2021). Secretory cells dominate airway CFTR expression and function in human airway superficial epithelia. Am. J. Respir. Crit. Care Med. 203, 1275–1289. 10.1164/rccm.202008-3198OC 33321047 PMC8456462

[B72] Park H. Shin D. H. Sim J.-R. Aum S. Lee M. G. (2020). IRE1α kinase–mediated unconventional protein secretion rescues misfolded CFTR and pendrin. Sci. Adv. 6, eaax9914. 10.1126/sciadv.aax9914 32128399 PMC7030921

[B73] Peter K. Varga K. Bebok Z. McNicholas-Bevensee C. M. Schwiebert L. Sorscher E. J. (2002). Ablation of internalization signals in the carboxyl-terminal tail of the cystic fibrosis transmembrane conductance regulator enhances cell surface expression. J. Biol. Chem. 277, 49952–49957. 10.1074/jbc.M209275200 12376531

[B74] Plasschaert L. W. Žilionis R. Choo-Wing R. Savova V. Knehr J. Roma G. (2018). A single-cell atlas of the airway epithelium reveals the CFTR-rich pulmonary ionocyte. Nature 560, 377–381. 10.1038/s41586-018-0394-6 30069046 PMC6108322

[B75] Pless D. D. Lennarz W. J. (1977). Enzymatic conversion of proteins to glycoproteins. Proc. Natl. Acad. Sci. 74, 134–138. 10.1073/pnas.74.1.134 264667 PMC393212

[B76] Poulsen J. H. Fischer H. Illek B. Machen T. E. (1994). Bicarbonate conductance and pH regulatory capability of cystic fibrosis transmembrane conductance regulator. Proc. Natl. Acad. Sci. 91, 5340–5344. 10.1073/pnas.91.12.5340 7515498 PMC43990

[B77] Prince L. S. Peter K. Hatton S. R. Zaliauskiene L. Cotlin L. F. Clancy J. P. (1999). Efficient endocytosis of the cystic fibrosis transmembrane conductance regulator requires a tyrosine-based signal. J. Biol. Chem. 274, 3602–3609. 10.1074/jbc.274.6.3602 9920908

[B78] Roxo-Rosa M. Xu Z. Schmidt A. Neto M. Cai Z. Soares C. M. (2006). Revertant mutants G550E and 4RK rescue cystic fibrosis mutants in the first nucleotide-binding domain of CFTR by different mechanisms. Proc. Natl. Acad. Sci. 103, 17891–17896. 10.1073/pnas.0608312103 17098864 PMC1693843

[B79] Saha S. Das A. Patra C. Anilkumar A. A. Sil P. Mayor S. (2022). Active emulsions in living cell membranes driven by contractile stresses and transbilayer coupling. Proc. Natl. Acad. Sci. 119, e2123056119. 10.1073/pnas.2123056119 35867835 PMC9335261

[B80] Santos J. D. Pinto F. R. Ferreira J. F. Amaral M. D. Zaccolo M. Farinha C. M. (2020). Cytoskeleton regulators CAPZA2 and INF2 associate with CFTR to control its plasma membrane levels under EPAC1 activation. Biochem. J. 477, 2561–2580. 10.1042/BCJ20200287 32573649

[B81] Sato Y. Kim D. Turner M. J. Luo Y. Zaidi S. S. Z. Thomas D. Y. (2023). Ionocyte-specific regulation of cystic fibrosis transmembrane conductance regulator. Am. J. Respir. Cell Mol. Biol. 69, 281–294. 10.1165/rcmb.2022-0241OC 36952679

[B82] Sato Y. Mustafina K. R. Luo Y. Martini C. Thomas D. Y. Wiseman P. W. (2021). Nonspecific binding of common anti-CFTR antibodies in ciliated cells of human airway epithelium. Sci. Rep. 11, 23256. 10.1038/s41598-021-02420-x 34853321 PMC8636639

[B83] Scudieri P. Musante I. Venturini A. Guidone D. Genovese M. Cresta F. (2020). Ionocytes and CFTR chloride channel expression in normal and cystic fibrosis nasal and bronchial epithelial cells. Cells 9, 2090. 10.3390/cells9092090 32933106 PMC7565890

[B84] Shen X. Ellis R. E. Lee K. Liu C. Y. Yang K. Solomon A. (2001). Complementary signaling pathways regulate the unfolded protein response and are required for *C. elegans* development. Cell 107, 893–903. 10.1016/S0092-8674(01)00612-2 11779465

[B85] Sheppard D. N. Welsh M. J. (1999). Structure and function of the CFTR chloride channel. Physiol. Rev. 79, S23–S45. 10.1152/physrev.1999.79.1.S23 9922375

[B86] Short D. B. Trotter K. W. Reczek D. Kreda S. M. Bretscher A. Boucher R. C. (1998). An apical PDZ protein anchors the cystic fibrosis transmembrane conductance regulator to the cytoskeleton. J. Biol. Chem. 273, 19797–19801. 10.1074/jbc.273.31.19797 9677412

[B87] Silvis M. R. Bertrand C. A. Ameen N. Golin-Bisello F. Butterworth M. B. Frizzell R. A. (2009). Rab11b regulates the apical recycling of the cystic fibrosis transmembrane conductance regulator in polarized intestinal epithelial cells. Mol. Biol. Cell 20, 2337–2350. 10.1091/mbc.e08-01-0084 19244346 PMC2669039

[B88] Swiatecka-Urban A. Boyd C. Coutermarsh B. Karlson K. H. Barnaby R. Aschenbrenner L. (2004). Myosin VI regulates endocytosis of the cystic fibrosis transmembrane conductance regulator. J. Biol. Chem. 279, 38025–38031. 10.1074/jbc.M403141200 15247260

[B89] Swiatecka-Urban A. Brown A. Moreau-Marquis S. Renuka J. Coutermarsh B. Barnaby R. (2005). The short apical membrane half-life of rescued {Delta}F508-cystic fibrosis transmembrane conductance regulator (CFTR) results from accelerated endocytosis of {Delta}F508-CFTR in polarized human airway epithelial cells. J. Biol. Chem. 280, 36762–36772. 10.1074/jbc.M508944200 16131493

[B90] Swiatecka-Urban A. Duhaime M. Coutermarsh B. Karlson K. H. Collawn J. Milewski M. (2002). PDZ domain interaction controls the endocytic recycling of the cystic fibrosis transmembrane conductance regulator. J. Biol. Chem. 277, 40099–40105. 10.1074/jbc.M206964200 12167629

[B91] Swiatecka-Urban A. Talebian L. Kanno E. Moreau-Marquis S. Coutermarsh B. Hansen K. (2007). Myosin vb is required for trafficking of the cystic fibrosis transmembrane conductance regulator in rab11a-specific apical recycling endosomes in polarized human airway epithelial cells. J. Biol. Chem. 282, 23725–23736. 10.1074/jbc.M608531200 17462998

[B92] Travaglini K. J. Krasnow M. A. (2018). Profile of an unknown airway cell. Nature 560, 313–314. 10.1038/d41586-018-05813-7 30097657

[B93] Travaglini K. J. Nabhan A. N. Penland L. Sinha R. Gillich A. Sit R. V. (2020). A molecular cell atlas of the human lung from single-cell RNA sequencing. Nature 587, 619–625. 10.1038/s41586-020-2922-4 33208946 PMC7704697

[B94] Tümmler B. (2023). Puzzle resolved: CFTR mediates chloride homeostasis by segregating absorption and secretion to different cell types. J. Clin. Invest. 133, e174667. 10.1172/JCI174667 37843282 PMC10575718

[B95] Turner M. J. Abbott-Banner K. Thomas D. Y. Hanrahan J. W. (2021). Cyclic nucleotide phosphodiesterase inhibitors as therapeutic interventions for cystic fibrosis. Pharmacol. Ther. 224, 107826. 10.1016/j.pharmthera.2021.107826 33662448

[B96] Varga K. Jurkuvenaite A. Wakefield J. Hong J. S. Guimbellot J. S. Venglarik C. J. (2004). Efficient intracellular processing of the endogenous cystic fibrosis transmembrane conductance regulator in epithelial cell lines. J. Biol. Chem. 279, 22578–22584. 10.1074/jbc.M401522200 15066992

[B97] von Heijne G. (1985). Signal sequences: the limits of variation. J. Mol. Biol. 184, 99–105. 10.1016/0022-2836(85)90046-4 4032478

[B98] Wang X. Matteson J. An Y. Moyer B. Yoo J.-S. Bannykh S. (2004). COPII-dependent export of cystic fibrosis transmembrane conductance regulator from the ER uses a di-acidic exit code. J. Cell Biol. 167, 65–74. 10.1083/jcb.200401035 15479737 PMC2172508

[B99] Ward C. L. Kopito R. R. (1994). Intracellular turnover of cystic fibrosis transmembrane conductance regulator. Inefficient processing and rapid degradation of wild-type and mutant proteins. J. Biol. Chem. 269, 25710–25718. 10.1016/S0021-9258(18)47306-1 7523390

[B100] Yoo J. S. Moyer B. D. Bannykh S. Yoo H. M. Riordan J. R. Balch W. E. (2002). Non-conventional trafficking of the cystic fibrosis transmembrane conductance regulator through the early secretory pathway. J. Biol. Chem. 277, 11401–11409. 10.1074/jbc.M110263200 11799116

[B101] Yuan F. Gasser G. N. Lemire E. Montoro D. T. Jagadeesh K. Zhang Y. (2023). Transgenic ferret models define pulmonary ionocyte diversity and function. Nature 621, 857–867. 10.1038/s41586-023-06549-9 37730992 PMC10533402

[B102] Zerangue N. Schwappach B. Jan Y. N. Jan L. Y. (1999). A new ER trafficking signal regulates the subunit stoichiometry of plasma membrane KATP channels. Neuron 22, 537–548. 10.1016/S0896-6273(00)80708-4 10197533

[B103] Zhao Y. Cushing P. R. Smithson D. C. Pellegrini M. Pletnev A. A. Al-Ayyoubi S. (2018). Cysteine modifiers suggest an allosteric inhibitory site on the CAL PDZ domain. Biosci. Rep. 38, BSR20180231. 10.1042/BSR20180231 29472314 PMC6435542

